# Health perceptions of patients with forgotten double-J stent

**DOI:** 10.7717/peerj.18156

**Published:** 2024-10-09

**Authors:** Mehmet Sezai Ogras, Kadir Yildirim

**Affiliations:** 1 Urology, Health Science University, Elazig, Turkey; 2 Urology, Medical Park Hospital, Elazig, Turkey

**Keywords:** Health, Perceptions, Forgotten, DJS

## Abstract

**Aim:**

In this article, we aimed to determine the health perceptions of the patients who did not apply for double-J stent (DJS) removal on time and evaluate whether health perceptions impact this behavior.

**Materials and Methods:**

Health perceptions of 22 patients who were treated for forgotten double-J stent (FDJS) between January 2017–October 2020 and 25 patients who applied for DJS removal during the given appointment time in the urology department of Elazig Training and Research Hospital and Elazig Fethi Sekin City Hospital were compared using the “Health Perception Scale”.

**Results:**

Health perception scale scores in the FDJS group and control group were 62-68 (mean = 64.27 ± 1.75) and 63-68 (mean = 65.36 ± 1.55), respectively. With regard to the sub-factors of the scale, control center scores were 21.86 ± 0.77 and 22.08 ± 1.15; precision scores were 16.54 ± 0.80 and 16.56 ± 0.82; importance of health scores were 12.77 ± 0, 92 and 13.32 ± 0.74; and self-awareness points were 13.04  ± 0.72 and 13.48 ± 0.58, respectively. There was a statistically significant difference between the health perception scale in total, importance of health, and self-awareness scores between the two groups. At the same time, there was no difference in terms of the control center and precision scores (*p*∗ = 0.029, *p*∗ = 0.030, *p*∗ = 0.028, *p* = 0.460, *p* = 0.951).

**Conclusion:**

Patients’ and their families’ educational status, income levels and lack of follow-up by healthcare professionals play an important role in forgotten DJS cases and patients’ perception of health may also have an impact on this behavior.

## Introduction

Double-J stents (DJSs) are often placed temporarily in several urological procedures, especially for renal, ureteral, urogynecological, or retroperitoneal pathologies that lead to obstruction, before shockwave lithotripsy, to reduce the risk of iatrogenic trauma before abdominal surgery, to provide passage during surgery following ureter injuries and during urological surgeries (Kuno et al., 1998). The duration of a DJS depends on the indications for implantation and generally varies between 2 to 12 weeks (Ray et al., 2015). Patients with DJS sign a form and are given an appointment for DJS removal at a specified time and the information about unwanted situations that may occur is also given. While the vast majority of patients apply to clinics for DJS removal at a specified time, approximately 12% do not come for this procedure (Mohammad et al., 2024). The educational status of the patients and their families and the lack of counseling or follow-up by health professionals play an important role in forgotten DJS (FDJS) cases. Although patients are aware of the severe complications that may occur, this behavior of not attending the specified appointment for the stent removal may be related to the health perceptions or behaviors of the people. Many conceptual models, such as the health belief model and social learning model have been developed to clarify the formation of health behaviors that affect the health status of the individual and how these behaviors can be altered. The Health Belief Model (HBM) and Social Learning Model (Social Cognitive Theory) are key frameworks in understanding health behavior. The HBM, developed in the 1950s, posits that health actions are driven by personal beliefs about susceptibility, severity, benefits, and barriers, with cues to action and self-efficacy also playing vital roles. On the other hand, Social Learning Theory, introduced by Albert Bandura, highlights the role of observational learning, reciprocal determinism, and self-efficacy in behavior change. While the HBM focuses on individual health decisions, Social Learning Theory emphasizes the influence of social interactions and environmental factors in shaping behavior. Both models are instrumental in designing effective health interventions (Bandura, 1986; Rosenstock, 1974; Glanz, Rimer & Viswanath, 2015a; Glanz, Rimer & Viswanath, 2015b). In these models, perceptions, beliefs, and attitudes are stated as factors affecting health behaviors (Diamond et al., 2007). Individuals’ health perceptions regarding their health are very important because they affect their behaviors, understanding and approaches to health problems (Ostwald et al., 1999). In our study, we investigated whether perceptions, attitudes and beliefs had an impact on forgotten DJS by using the “Perception of Health Scale” developed by Diamond et al. (2007).

## Materials and Methods

After obtaining the approval of Firat University Non-Invasive Research Ethics Committee dated 04.12.2020 and numbered 2020/16-16, twenty-two patients presenting with severe urological symptoms who were treated for FDJS in the urology department of Elazig Training and Research Hospital and Elazig Fethi Sekin City Hospital between January 2017 and October 2020. Twenty-five patients (control group) who applied for DJS removal within the specified time were invited to the clinic. The files of patients who applied to our clinic for DJS removal for various indications between January 2017 and December 2020 were retrospectively scanned. It was determined that 27 of 523 patients whose DJS catheters were removed between these dates had exceeded 180 days from the date of DJS application. Of these patients, five patients who had a history of psychiatric drug use, had neurological diseases, and were under the age of 18 were not included in the study. The “Perception of Health Scale” questionnaire, a new five-point Likert-type scale with 15 questions developed by Diamond et al. (2007), was given to the patients who came to the clinic. Questionnaire questions were asked on the phone to the patients who could not come, and the results were recorded. The results were evaluated using the SPSS 17.0 package program.

## Results

A total of 22 patients with forgotten DJS were detected. In our clinic, double-J stents were placed in 523 patients for different indications. The patients had symptoms such as hematuria, irritative voiding symptoms, and flank pain but had not sought medical attention for a long time. They presented to our center when their symptoms worsened. Also, some patients were noticed while being examined for different reasons and were referred to our clinic.

Table 1 Patients presenting due to DJS had their age (year), sex, stent indications, indwelling time (day), location of encrustation, procedures, presenting symptoms and perception of health scale.

In the FDJS group, three (13.6%) of the patients were female and 19 (86.4%) were male. Their ages varied between 19 and 72 years. Their average age was 40.27 ± 15.26 years, the duration of DJS was between 210–1.980 days, the average duration was 628.27 ± 459.48 days, and the health perception scale scores were between 62-68, and the average score was 64.27 ± 1.75. In the control group, five (20%) of the 25 patients were female, 20 (80%) were male, their ages were between 19 and 71 years and their mean age was 37.32 ± 8.25 years. The duration of DJS was between 18 and 56 days, the average duration was 28.4 ± 8.25 days, and the health perception scale scores ranged from 63 to 68, with an average of 65.36 ± 1.55 (Table 2). There was a significant difference between the total health perception scores (p* = 0.029). When the sub-factors of the scale were compared, the control center scores were 21.86 ± 0.77 in the FDJS group and 22.08 ± 1.15 in the control group, there was no statistically significant difference between them (*p* = 0.460). Mean precision score was 16.54 ± 0.80 points in the FDJS group and 16.56 ± 0.82 in the control group, there was no statistically significant difference between the groups (*p* = 0.951). The importance of health score was 12.77 ± 0.92 in the FDJS group and 13.32 ± 0.74 in the control group, there was a statistically significant difference between the groups (p* = 0.030). Self-awareness score was 13.04 ± 0.72 in the FDJS group and 13.48 ± 0.58 in the control group. There was a statistically significant difference between the groups. (p* = 0.028) (Table 3). Also, when both groups were evaluated, there was a negative correlation between age and health perception scale (p* = 0.002).

**Table 1:** Patients, age, duration of the stent, total health perception scale scores.

**No**	**Age year**	**sex**	**Stent indications**	**Indwelling time (day)**	**Location of encrustation**	**Procedures**	**Presenting symptoms**	**Perception of health scale**
1	66	M	Abdominal Surgery	1,980	Kidney, urether bladder	URS, PNL, Open cystolithotomi	Hematuria, irritative voiding symptom	46
2	76	M.	Abdominal Surgery	1,586	Kidney, urether bladder	URS, PCNL, EnCL	Irritative voiding symptom	48
3	54	M	PCNL	1,213	Kidney, urether bladder	SWL, URS, RIRS, EnCL	Loin pain, irritative voiding symptom	50
4	27	F	URS	485	Kidney	SWL	Irritative voiding symptom	52
5	33	F	SWL	387	Kidney, urether bladder	SWL, URS, RIRS, EnCL	Irritative voiding symptom	47
6	22	M	Piyeloplasti	456	–	SCSR	–	55
7	36	M	PCNL	612	Kidney, bladder	SWL, EnCL	Loin pain, hematuria	52
8	29	F	Pregnancy	210	Bladder, urether	URS, EnCL	Fever, Irritative voiding symptom	56
9	43	M	URS	624	Urether, bladder	URS, EnCL	Irritative voiding symptom	48
10	63	M	PCNL	1,254	Kidney, urether bladder	PNL, URS, EnCL	Loin pain, irritative voiding symptom	46
11	35	M	URS	413	Urether, bladder	URS	Irritative voiding symptom	53
12	37	M	URS	382	Urether	URS	–	52
13	26	F	SWL	374	Kidney	PNL, URS, EnCL	Hematuria, loin pain	56
14	33	M	Trauma	542	Bladder	EnCL	Irritative voiding symptom	47
15	43	M	PCNL	324	Kidney, urether, bladder	PNL, URS, EnCL	Nausea and vomiting	52
16	48	M	Abdominal Surgery	474	Urether	URS	–	45
17	19	M	URS	396	Urether	URS, RIRS	–	47
18	48	M	PCNL	549	Kidney, urether bladder	SWL, URS, EnCL, RIRS	Hematuria	61
19	34	F	URS	336	Urether bladder	URS, EnCL	Irritative voiding symptom, loin pain	52
20	42	M	PCNL	223	–	SCSR	–	45
21	26	M	URS	370	Urether	URS, RIRS, EnCL	Irritative voiding symptom	48
**22**	56	M	URS	632	Kidney, Urether	PNL, URS	Loin pain, fever	45

**Notes.**

URS Ureterorenoscopy RIRS Retrograde Intrarenal Surgery PCNL Percutaneous Nephrotolithotomy SWL Shock Wave Lithotripsy EnCL Endoscopic cystolithotomy SCSR Simple Cystoscopic Stent Removal

**Table 2:** Number, age, duration of the stent, total health perception scale scores.

	Number	Age (year)	Duration of the stent (days)	Perception of health scale
FDJS group	22	40.27 ± 15.26	628.27 ± 459.48	64.27 ± 1.75
Control group	25	37.32 ± 8.25	28.4 ± 8.25	65.36 ± 1.55
*p* value				*p*∗ = 0.029

**Table 3:** The perception of health sub-dimension scores of patients.

	Control center	Precision	Importance of health	Self-Awareness
FDJS group	21.86 ± 0.77	16.54 ± 0.80	12.77 ± 0.92	13.04 ± 0.72
Control group	22.08 ± 1.15	16.56 ± 0.82	13.32 ± 0.74	13.48 ± 0.58
*p* value	*p* = 0.460	*p* = 0.951	*p*∗ = 0.030	*p*∗ = 0.028

## Discussion

Forgotten double-J stents (FDJSs) are still a significant clinical problem today. DJSs have been used in urology practice since 1967, especially for renal, ureteral, urogynecological, an retroperitoneal pathologies, shockwave lithotripsy (SWL), to reduce the risk of iatrogenic trauma before abdominal surgery or to provide passage during urological operations and following ureter injuries that occurred due to surgery (Kuno et al., 1998; Kadıoğlu & Yildiz, 2012). They are often inserted temporarily. Depending on the reason for use, the duration of the stent generally varies between 2 to 12 weeks (Ray et al., 2015). In practice, there is not a strict time frame universally defining a forgotten stent, but most cases label stents as forgotten after they have exceeded the manufacturer’s recommended safe limit or have stayed in place for 6–12 months unintentionally (Patil et al., 2020). We included forgotten DJS that remained in place for more than 180 days in our study. Although an appointment to remove the stent is made at the time of discharge and patients are informed about the complications that may occur if they do not attend their appointment, approximately 12% of the DJSs are forgotten (Mohammad et al., 2024). In our study, the rate of patients with FDJS was determined as 5.1%. This rate is consistent with the literature (Patil et al., 2020). DJSs may stay longer than they should due to patient and/or non-patient negligence. The education status of patients and families lack of counseling or follow-up provided by healthcare professionals play an important role in forgotten DJS cases (Devraj et al., 2016). In our study, the educational level and income level were different between both groups (Table 4).

**Table 4:** Education and income levels of patients.

	**Education (years)**				**Income**			
	0–5	6–8	9–12	12	Low	Middle low	Middle high	High
FDJS group	15	5	2	–	16	5	1	–
Control group	–	2	14	8	1	5	17	2

**Figure 1:**
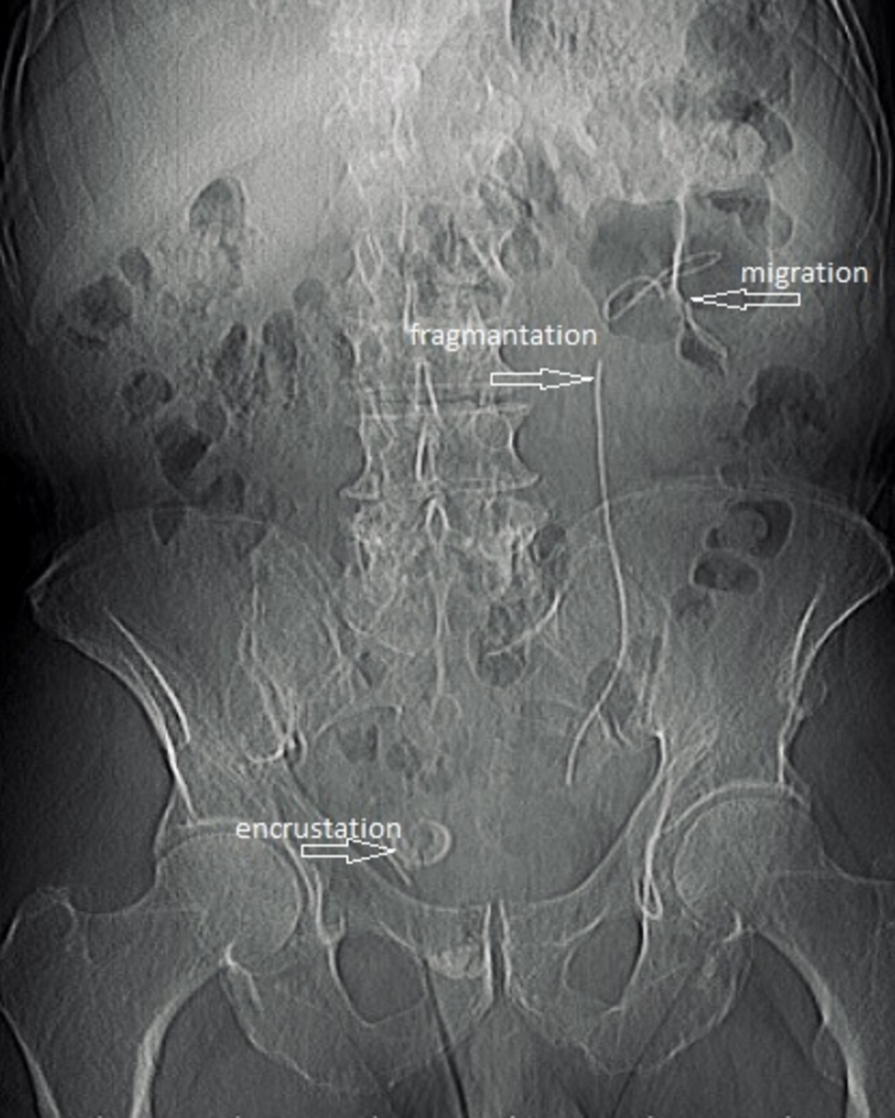
Migration, encrustation, fragmentation in FDJS.

FDJSs can lead to complications such as recurrent infection, migration, encrustation, fragmentation, sepsis, and renal failure (Damiano et al., 2002; Kawahara et al., 2012) (Fig. 1). When complications due to FDJS occur, patients should be evaluated appropriately, and the most suitable treatment method should be applied. While some FDJSs require a simple procedure such as simple cystoscopic stent removal under local anesthesia, for the majority of them, shockwave lithotripsy, percutaneous nephrolithotomy, ureterorenoscopy, retrograde intrarenal surgery, endoscopic cystolithotripsy, percutaneous cystolithotripsy, open cystolithotripsy or nephrectomy may be required alone or in combination (Adanur & Ozkaya, 2016). While the majority of patients with DJS apply to clinics at the specified time and come to their appointment, some patients never come for follow-up. Patients apply to urology clinics when complications related to DJS develop or coincidentally, they apply to urology clinics for another disease and DJS stent is detected during diagnostic work up. Despite their knowledge about the possible severe complications, patients’ negligence in attending specified follow-up appointments might be related to health perception or behavior. Many conceptual models, such as the health belief model and the social learning model, have been developed to clarify the development of health behaviors that affect the health status of the individual and how these behaviors can be altered. In these models, perceptions, attitudes and beliefs are stated as factors affecting health behaviors (Diamond et al., 2007). In our study, we used the 5-point Likert-type “Health Perception Scale” of which the original language was English, developed by Diamond et al. (2007) and adapted to Turkish by Kadıoğlu & Yıldız (2012). The scale has 15 items and four sub-dimensions: a control center consisting of five items, self-awareness consisting of three items, the importance of health consisting of three items, and precision consisting of four items. The response time is 5–7 min. We gave our patients a 7-minute response time. Negative statements in the scale are scored in reverse, and the scores that can be obtained from the scale vary between 15 and 75 points. In our study, the mean health scale score was calculated as 64.27 ± 1.75 in the FDJS group and 65.36  ± 1.55 in the control group. While there was no statistically significant difference between the FDJS group and the control group in terms of the sub-dimensions of the scale, control center and precision (*p* = 0.460, *p* = 0.951), there was a statistically significant difference between the total score of the health perception scale, the importance of health and self-awareness scores (p* = 0.029, p* = 0.030, p* = 0.028). These results show that perception of health may have an impact on forgotten DJS along with other causative factors. The way health is perceived is an indicator that enables individuals to self-assess their health, psychosocial and biological status (Turgut & Bekar, 2008). Similar to our study, Jylhä (2009) stated in her study that health perception is influenced by subjective health status, which represents an individual’s own assessment of their overall health. Similarly, Shin & Kim (2004) stated that this subjective perception of individuals’ health determines the attitudes that lead or do not lead to health behaviors. Individuals’ perceptions of their health are of great importance as they affect their behavior toward health problems, their understanding of problems and their approaches to these problems (Ostwald et al., 1999). Individuals’ perceptions of health differ. The reasons for these differences include personality and cognitive factors, perceived symptoms, emotional factors, demographic factors and social factors (Sakr et al., 2023). Lee & Chung (1998) stated that health perception is an important factor associated with health-promoting behaviors in older adults and shows strong connections with behaviors aimed at improving health. In another study, Kwak et al. (2022) Improving positive subjective health perception is vital for disease prevention and continued management of prediabetes and diabetes through healthy behaviors. Individuals with lower living standards and socioeconomic levels have found to have more negative perceptions of general health, and it has been found that as the age increases, the perception of health becomes negative (Lam & Gupta, 2002). In a study conducted by Lawrence & Schank (1993) no significant difference in health perception was found between nursing students and non-nursing students. Another study argued that health perception provides valuable insights into health status; however, this study did not demonstrate a relationship between health perception and health behavior (Han & Cho, 2001). Health perception scale scores were low among in our patients and there was a negative correlation between them (*p* = 0.002). Vajpeji et al. (2020) reported in their study that higher number of cases with forgotten DJS was observed in illiterate and low-income patients. The reason for this may be the low perception of health.

## Conclusion

Education status, income levels of patients and families, and lack of counseling or follow-up provided by health professionals play an essential role in forgotten DJS. The general health perceptions of individuals with lower living standards and socioeconomic levels are adversely affected, and one reason why individuals with lower socioeconomic status and living standards do not apply to clinics on time for DJS removal may be their lower perception of health.

##  Supplemental Information

10.7717/peerj.18156/supp-1 Supplemental Information 1 Raw data

## Additional Information and Declarations

### Data Availability

The following information was supplied regarding data availability: The raw measurements are available in the Supplementary File.
